# Properties of Blended Cement Containing Iron Tailing Powder at Different Curing Temperatures

**DOI:** 10.3390/ma15020693

**Published:** 2022-01-17

**Authors:** Heng Wang, Fanghui Han, Shaochang Pu, Hongbo Zhang

**Affiliations:** 1 Beijing Urban Construction Group Co., Ltd., Beijing 100081, China; wangheng@mail.bucg.com; 2 Beijing Key Laboratory of Urban Underground Space Engineering, Department of Civil Engineering, University of Science and Technology Beijing, Beijing 100083, China; g20198091@xs.ustb.edu.cn (S.P.); g20208156@xs.ustb.edu.cn (H.Z.)

**Keywords:** iron tailing powder, blended cement, hydration heat, microstructure, compressive strength

## Abstract

The properties of blended cement containing 0%, 20%, and 50% iron tailing powder (ITP) at 20 °C and 60 °C were investigated by determining the hydration heat, microstructure, and compressive strength. The addition of ITP decreases the exothermic rate and cumulative hydration heat of blended cement at 20 °C. The high temperature increases the hydration rate and leads to the hydration heat of blended cement containing 20% ITP higher than that of Portland cement. Increasing the amount of ITP decreases the non-evaporable water content and Ca(OH)_2_ content as well as compressive strength at both of the two studied temperatures. The addition of ITP coarsens the early-age pore structure but improves the later-age pore structure at 20 °C. The high temperature significantly improves the early-age properties of blended cement containing ITP, but it is detrimental to the later-age properties development. The reaction of ITP is limited even at high temperature. The large ITP particles bond poorly with surrounding hydration products under early high-temperature curing condition. The properties of blended cement containing a large amount of ITP are much poorer at high temperature.

## 1. Introduction

In order to meet the rapid development of society, many iron ores are mined in China. A considerable amount of iron tailings are discharged while extracting useful metals. The common method to deal with iron tailings is stockpiling, which has caused land occupation and surface subsidence as well as environmental pollution [1,2,3]. It is urgent to find an efficient way to improve the utilization rate of iron tailings. The main chemical compositions of iron tailings are SiO_2_, Al_2_O_3_, Fe_2_O_3_, CaO, and MgO [4]. Iron tailings show the same kind of chemical compositions as that of Portland cement. Thus, iron tailings can be used in the concrete industry.

Many studies have been conducted on the properties of concrete prepared with iron tailings as fine aggregate. The workability of concrete is slightly reduced with increasing iron tailings content as a result of higher specific gravity and larger water requirement [5,6]. However, the iron tailings concrete shows superior mechanical properties compared with the conventional concrete [7]. Replacing 40% manufactured sand with iron tailings makes the ultra-high performance concrete have the highest compressive strength [8]. The recycled concrete exhibits the high mechanical properties and compact microstructure when the content of iron tailings is 20–40% [9]. An appropriate substitution for iron tailings can result in satisfactory durability of concrete [1,9,10,11].

To obtain a greater amount of valuable metals, the enhanced crushing and grinding of iron ore leads to smaller particle sizes of iron tailings. The finer iron tailings become powder and is not suitable to be used as fine aggregate. It is feasible to utilize iron tailing powder (ITP) as a mineral admixture. Iron tailings is inert with nearly no activity. However, the mechanical grinding stimulates the activity of iron tailings [12,13]. The finer the ITP, the higher the activity. The ettringite and C-S-H gel are found in the ITP-Ca(OH)_2_-anhydrite system [14]. ITP belongs to pozzolanic materials with low activity [15], but its filler effect is significant. To ensure the properties of concrete, the replacement ratio of ITP should not be higher than 30% [12,16]. The concrete prepared with ITP and slag has satisfactory properties [17]. The compressive strength of mortar prepared with 30% Portland cement, 35% slag, 30% ITP, and 5% gypsum is higher than 45 MPa at 28 days [18]. The addition of ITP also improves the durability of concrete when the replacement ratio is no more than 30% [19,20]. The properties of concrete containing 40% ITP cured according to the adiabatic temperature rise curve were studied and it was found that the high-volume ITP concrete cured under the temperature match curing condition has poor durability [21]. In our previous published paper, the influences of fineness of ITP on hydration of the composite binder within 3 days at 20 °C were investigated [22]. The curing temperatures and long-term properties were not considered in the literature [22].

The use of ITP as a mineral admixture can solve the shortage of the traditional mineral admixtures, such as fly ash and slag. The addition of a mineral admixture can improve the workability, compressive strength, durability, and even shielding properties [23,24]. It is beneficial to the sustainable development of the concrete industry and the improvement of the use ratio of ITP. However, all current studies related to the properties of blended cement containing ITP are carried out at room temperature. Nearly no previous study has provided information on the influence of temperature on the properties of blended cement containing ITP. The hydration of Portland cement is an exothermic process. The low thermal conductivity of concrete increases the inner temperature. The reaction of blended cement containing ITP at the higher temperature must be considered. However, what remains unclear is the hydration mechanism and properties development of blended cement containing ITP at the high temperature. Therefore, in this paper, the hydration heat, non-evaporable water content, pore structure, Ca(OH)_2_ content, morphology, and compressive strength were investigated at different temperatures. Considering that the core temperature of massive concrete is about 60 °C, the high curing temperature is 60 °C in this study. The curing temperature under standard curing condition is 20 °C. Hence, 20 °C and 60 °C were selected to investigate the influences of curing temperatures. Considering that the small amount and larger amount of ITP are added to blended cement. The replacement ratios of ITP were 0%, 20%, and 50%. The primary aim of this paper is to critically analyze the influences of temperature on the properties of blended cement containing ITP.

## 2. Materials and Methods

### 2.1. Raw Materials

**Figure 1:**
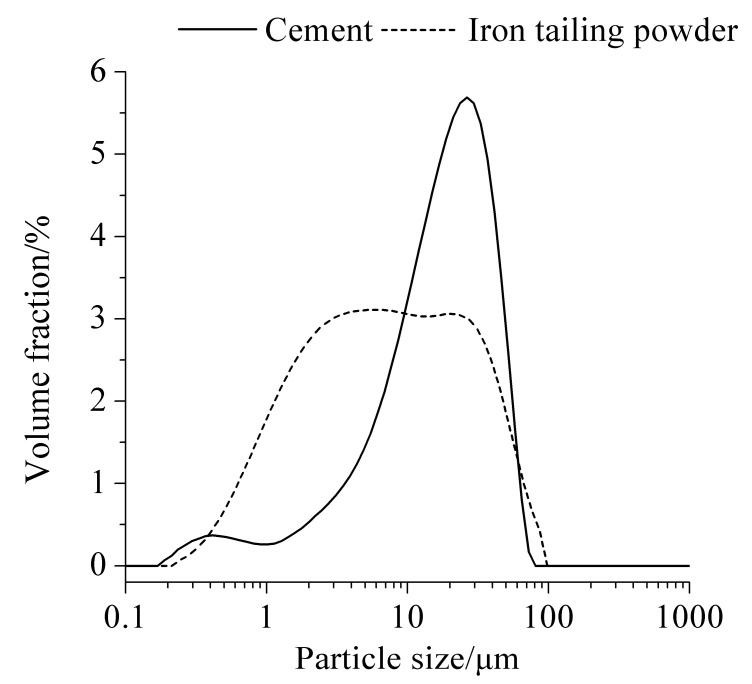
Particle size distribution of raw materials.

P.I 42.5 Portland cement and ITP obtained from a mining enterprise in Beijing were used in this investigation. The chemical compositions of Portland cement and ITP are given in Table 1. ITP has higher content of SiO_2_ and Al_2_O_3_ but lower content of CaO compared with Portland cement. The content of Fe_2_O_3_ is also high due to the ITP being produced from iron ore. Figure 1 presents the particle size distributions of Portland cement and ITP. The particle size of ITP is obviously smaller than that of Portland cement. ITP with particle size smaller than 10 μm accounts for more than 50%. A considerable amount of small ITP particles and some large ITP particles with angular shape were found in our previous study [22]. The water demand ratio of ITP was 104%. ISO standard sand was used to prepare the mortars. The SiO_2_ content, loss on ignition (LOI), and silt content of ISO standard sand are higher than 96%, no more than 0.40%, and no more than 0.20%, respectively. The cumulative residues of ISO standard sand through 0.65 mm, 0.40 mm, and 0.25 mm diameter square hole sieves are smaller than 3%, 40% ± 5%, and higher than 94%, respectively.

### 2.2. Mix Proportions

Table 2 and Table 3 display the mix proportions of pastes and mortars, respectively. Amounts of 0%, 20% and 50% Portland cement were replaced by ITP. The water/binder ratio was 0.4. An amount of 1% polycarboxylate superplasticizer was added to ensure the workability of the mortar. The prepared pastes were put into plastic tubes with 10 mL volume and then sealed. Mortars with dimensions of 40 × 40 × 160 mm were prepared. Half of the paste and mortar samples were cured under standard curing condition (20 °C, 95% relative humility). The other half of the samples were first cured at 60 °C for 7 days, after that, the samples were cured at 20 °C until the test ages.

### 2.3. Test Methods

The hydration heat of blended cement containing ITP was measured with a TAM air isothermal calorimeter produced by TA instruments in New Castle, America at 20 °C and 60 °C. The Portland cement, ITP, and water needed to be put in a place with a test temperature 24 h in advance. The weighed materials were mixed rapidly in a glass bottle and then the glass bottle was put into the channel of the isothermal calorimeter. The exothermic rate and the cumulative hydration heat of the blended cement containing ITP were determined continuously.

The hardened pastes were crushed into small pieces and soaked in ethanol at test ages. The non-evaporable water contents of cement-ITP hardened pastes were determined by high temperature ignition method. The non-evaporable water content was the mass loss between hardened paste at 80 °C and 1000 °C considering the loss on ignition of raw materials.

The pore structures of cement-ITP hardened pastes cured for 3 days and at 90 days were measured with a mercury injection porosimeter produced by Micromeritics company in Norcross, America. The maximum pressure was 300 MPa. The differential pore volume and the cumulative pore volume were obtained.

The properties of cement-ITP hardened pastes cured for 365 days were investigated with a thermalgravimetric analyzer produced by TA instruments in Shanghai, China, which was conducted on about 20 milligrams of hardened paste for each sample from room temperature to 900 °C under N_2_ protection. The heating rate is 10 °C min^−1^. The content of Ca(OH)_2_ was calculated based on the results of weight loss of Ca(OH)_2_ and CaCO_3_.

The backscattered electron (BSE) images of cement-ITP hardened pastes cured for 365 days were determined with a scanning electron microscope produced by FEI company in Eindhoven, Netherlands. The sample preparation process and the testing process were the same as those described in the literature [25].

The compressive strengths of cement-ITP mortars cured for 3 days, 7 days, 28 days, 90 days, and 365 days were tested.

## 3. Results and Discussion

### 3.1. Hydration Heat

**Figure 2:**
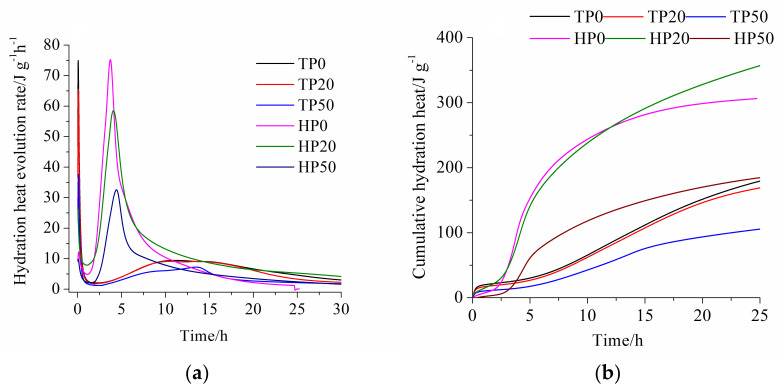
Hydration heat of blended cement containing ITP at 20 °C and 60 °C: (**a**) exothermic rate; (**b**) cumulative hydration heat.

The results of hydration heat of blended cement containing ITP at 20 °C and 60 °C are set out in Figure 2. From Figure 2a we can see that the exothermic rate curve of the blended cement containing 20% ITP (sample TP20) almost overlaps with that of Portland cement in the acceleration period at 20 °C. It is attributed to the much higher specific surface area of ITP. The surface of ITP provides additional nucleation sites for the growth of hydration products of cement. ITP is inert in the early reaction process at 20 °C [22]. The increased water/cement ratio further accelerates the reaction of cement [26,27]. The findings indicate that the addition of a small quantity of ITP does not affect the hydration of cement before the deceleration period at 20 °C. The exothermic rate of the blended cement containing 20% ITP is lower than that of Portland cement after about 20 h. When 50% Portland cement is substituted by ITP, the exothermic rate is obviously lower than that of Portland cement at 20 °C. Increasing temperature to 60 °C dramatically increases the exothermic rate of the blended cement containing ITP. The ending time of the induction period and the appearing time of the second exothermic peak both significantly shorten. Note that the exothermic rate of the blended cement containing 20% ITP (sample HP20) is higher in the induction period and the initial acceleration period compared with Portland cement. After that, it becomes lower. However, sample HP20 shows slightly higher exothermic rate in the deceleration period and the apparently higher exothermic rate in the steady period. It is indicated that the high temperature has stronger promoting effect on the early stage hydration of Portland cement and the later stage hydration of blended cement containing 20% ITP. Although the high temperature also significantly accelerates the hydration of the blended cement containing 50% ITP, the exothermic rate is still much lower. The high temperature has limited promoting effect on the blended cement containing a large amount of ITP. It is related to the low reactivity of ITP.

As can be seen from Figure 2b, the cumulative hydration heat of the blended cement containing 20% ITP is slightly lower than that of Portland cement at 20 °C. The cumulative hydration heat of the blended cement containing 50% ITP is evidently lower than that of Portland cement at 20 °C. The gap between the exothermic rate of the blended cement and that of Portland cement becomes larger with increasing time. Note that the decreasing ratio of the cumulative hydration heat is lower than the substitution ratio of ITP, which confirms that the addition of ITP promotes the hydration of the blended cement. The cumulative hydration heat of the blended cement containing ITP increases dramatically at 60 °C. The cumulative hydration heat of the blended cement containing 20% ITP exceeds that of Portland cement after about 12 h, and the subsequent increasing trend is still significant. The rapid reaction of the Portland cement is harmful to the later-age hydration due to the formation of thick hydrates around the unhydrated cement particles at 60 °C [28,29]. The filler effect of ITP is in favor of cement hydration at high temperature. More importantly, the high temperature might stimulate the reactivity of ITP. The reaction of ITP also contributes to the increase in hydration heat. However, the blended cement containing 50% ITP still has lower cumulative hydration heat compared with Portland cement at 60 °C. The reduction in Portland cement plays an important role during the hydration of blended cement. The results indicate that the high replacement ratio of ITP can dramatically decrease the early hydration rate even at the high temperature.

### 3.2. Non-Evaporable Water Content

**Figure 3:**
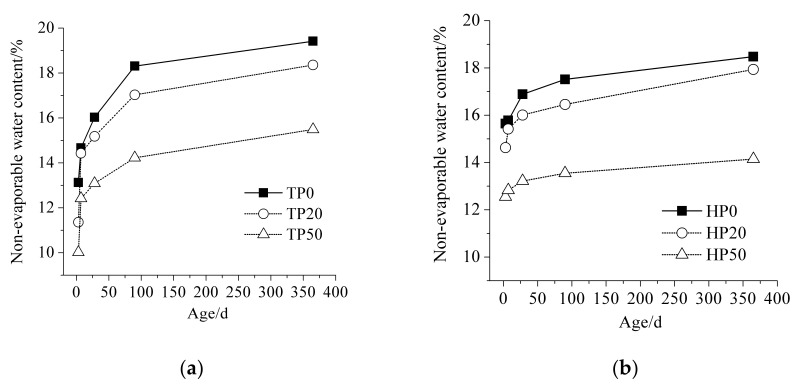
Non-evaporable water content of cement-ITP hardened paste at different curing temperatures: (**a**) 20 °C; (**b**) 60 °C.

Figure 3 illustrates the non-evaporable water content of cement-ITP hardened paste at different curing temperatures. As shown in Figure 3a, the non-evaporable water content decreases with increases in the amount of ITP at 20 °C. As aforementioned, ITP has very low reactivity. The early-age non-evaporable water content mainly comes from the hydration products of cement. The addition of ITP decreases the amount of Portland cement. In spite of accelerating the early hydration of cement by adding ITP, the cumulative hydration heat of blended cement containing ITP is still lower than that of Portland cement (Figure 2b). As a consequence, the generation of a small amount of hydration products leads to low non-evaporable water content for cement-ITP hardened paste. The increasing rate of non-evaporable water content of cement-ITP hardened paste from 3 days to 7 days is significantly high. However, the cement-ITP hardened paste still shows lower non-evaporable water content compared with Portland cement paste at later ages. It confirms that the amount of hydration products of cement-ITP hardened paste is still small after a long time curing at 20 °C.

Looking at Figure 3b, the early-age non-evaporable water contents of all hardened pastes at 60 °C are distinctly higher than that at 20 °C. The non-evaporable water contents of hardened pastes cured for 3 days at 60 °C are just slightly lower than that cured for 28 days at 20 °C. The high temperature increases the early hydration rate of blended cement containing ITP and more hydration heat releases (Figure 2). The rapid hydration produces abundant hydration products in a short time, and then the higher non-evaporable water content is determined at early age. However, the lower increasing rate of non-evaporable water content is observed after 28 days, especially for hardened paste containing 50% ITP. The non-evaporable water content of hardened paste containing 50% ITP has almost no increase from 90 days to 365 days at 60 °C. The growth and the morphology of the C-S-H gel are very sensitive to high temperature [30]. The increased apparent density of the C-S-H gel leads to the lower non-evaporable water content of hardened paste containing ITP at 60 °C compared with that at 20 °C. Note that the gap between the non-evaporable water content of the hardened paste containing 20% ITP and that of Portland cement paste becomes smaller at 60 °C. The non-evaporable water content of the hardened paste containing 20% ITP cured for 365 days is slightly lower than that of Portland cement paste at 60 °C. It might be due to the stimulating effect of high temperature on the reaction of ITP.

### 3.3. Pore Structure

**Figure 4:**
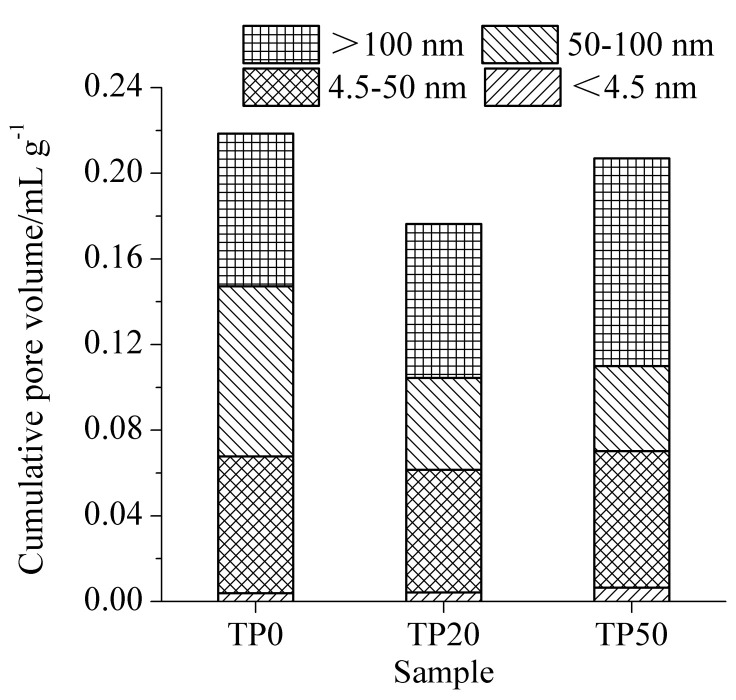
Pore structure of cement-ITP hardened paste cured for 3 days at 20 °C.

**Figure 5:**
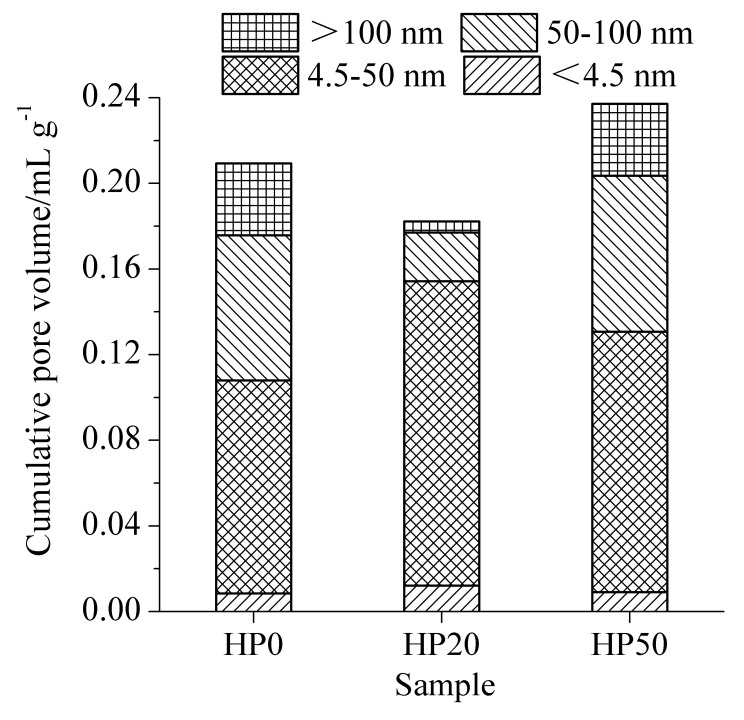
Pore structure of cement-ITP hardened paste cured for 3 days at 60 °C.

The results of pore structures of cement-ITP hardened paste cured for 3 days at 20 °C and 60 °C are presented in Figure 4 and Figure 5, respectively. Mehta [31] divided the pores in hardened paste into four grades based on the pore size: <4.5 nm, 4.5~50 nm, 50~100 nm, and >100 nm. The pores with pore size larger than 50 nm have a greater influence on the strength and permeability, while the pores with pore size smaller than 50 nm mainly affect the drying shrinkage and creep. As shown in Figure 4, it is apparent that the cumulative pore volume of hardened paste containing ITP is lower than that of Portland cement paste. It is related to the filler effect of ITP. The addition of ITP obviously reduces the content of pores with pore size of 50~100 nm. However, the content of large pores (pore diameter >100 nm) is clearly higher, especially for the hardened paste containing 50% ITP. The larger pores (>100 nm) significantly weaken the properties of concrete [32]. It confirms that the addition of a large amount of ITP is detrimental to the early-age pore structure of hardened paste. It is attributed to the extremely low activity of ITP at early age [22]. The slow hydration rate (Figure 2a) and the low content of hydration products (Figure 3a) results in the coarse pore structure. Increasing the temperature to 60 °C evidently decreases the cumulative pore volume of the Portland cement paste at 3 days (Figure 5). The promoting effect of the high temperature on early hydration of cement is significant. The cumulative pore volume of sample HP20 is significantly smaller than that of sample HP0. Furthermore, for sample HP20, the contents of <4.5 nm and 4.5~50 nm pores increase and the contents of 50~100 nm and >100 nm pores obviously decrease compared with sample HP0. It is indicated that the addition of a small amount of ITP is favorable to refinement of early pore structure at 60 °C. The cumulative pore volume of sample HP50 becomes larger than that of sample HP0 at 60 °C. The content of 4.5~50 nm pores is only increased compared with sample HP0. The contents of 50~100 nm and >100 nm pores of sample HP50 are almost identical to those of sample HP0. Although the cumulative pore volume of cement-ITP hardened paste at 60 °C is slightly higher than that at 20 °C, the contents of large pores (50~100 nm and >100 nm) are significantly reduced at 60 °C.

**Figure 6:**
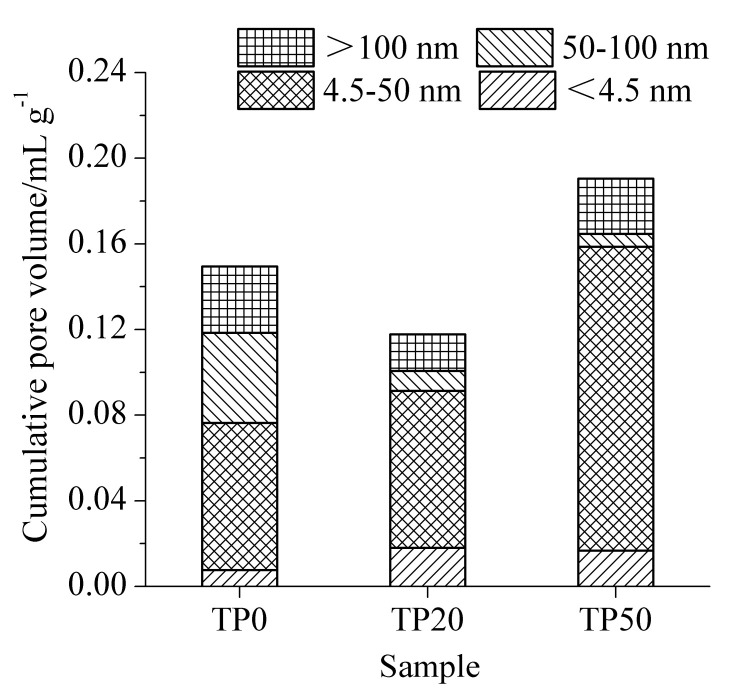
Pore structure of cement-ITP hardened paste cured for 365 days at 20 °C.

**Figure 7:**
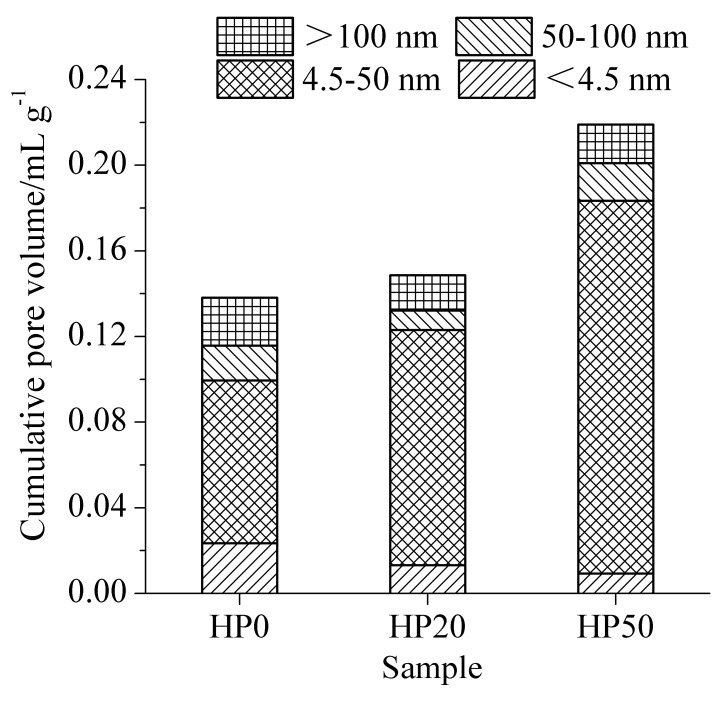
Pore structure of cement-ITP hardened paste cured for 365 days at 60 °C.

The results of pore structures of cement-ITP hardened paste cured for 365 days at 20 °C and 60 °C are presented in Figure 6 and Figure 7, respectively. As seen in Figure 6, the cumulative pore volume of hardened paste containing 20% ITP cured for 365 days is smaller compared with Portland cement paste at 20 °C. Adding 20% ITP markedly reduces the contents of large pores (50~100 nm and >100 nm) and increases the contents of small pores (<4.5 nm). The cumulative pore volume of sample TP50 is higher than that of sample TP0, but the increased pore volume is caused by the pores with a pore diameter of 4.5~50 nm. The content of large pores (50~100 nm and >100 nm) evidently decreases, especially for 50~100 nm pores. The results indicate that the addition of a certain amount of ITP improves the pore structure of the hardened paste cured for a long age at 20 °C. This is due to the smaller particle size of ITP and the reaction of ITP at a later age. The reaction of ITP generates ettringite and C-S-H gel [14], which fill the pores and then refine the pore structure. As shown in Figure 7, the cumulative pore volume increases with increasing ITP content at 60 °C. The cumulative pore volume of sample HP50 is dramatically larger than that of Portland cement paste. The cumulative pore volume of cement-ITP hardened paste at 60 °C is much higher than that at 20 °C. The findings elucidate that the early high-temperature curing is detrimental to the refinement of the pore structure of cement-ITP hardened paste cured for a long age, especially for hardened paste containing a high quantity of ITP. Increasing the early curing temperature apparently coarsens the later-age pore structure of cement-ITP hardened paste (Figure 6 and Figure 7).

### 3.4. Thermogravimetric Analysis

**Figure 8:**
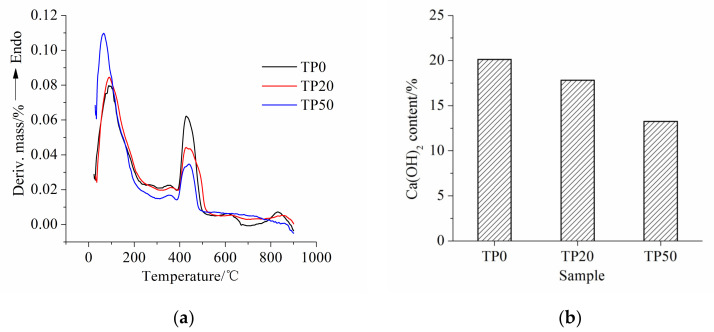
Thermogravimetric analysis of cement-ITP hardened paste cured for 365 days at 20 °C: (**a**) DTG curve; (**b**) Ca(OH)_2_ content.

**Figure 9:**
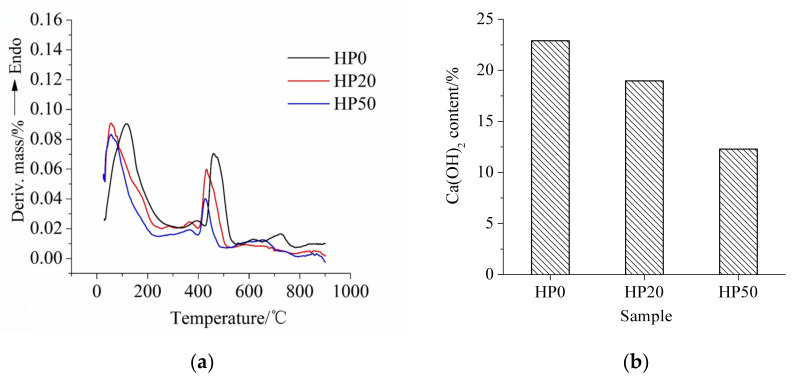
Thermogravimetric analysis of cement-ITP hardened paste cured for 365 days at 60 °C: (**a**) DTG curve; (**b**) Ca(OH)_2_ content.

The differential thermogravimetric (DTG) curve and Ca(OH)_2_ content of cement-ITP hardened paste cured for 365 days at 20 °C are set out in Figure 8. The endothermic peaks at 400~550 °C and 600~800 °C represent dewatering stages of Ca(OH)_2_ and CaCO_3_, respectively. From Figure 8a we can see that the endothermic peak of Ca(OH)_2_ notably becomes weak with increasing ITP content. The Ca(OH)_2_ contents calculated based on the thermogravimetric results are shown in Figure 8b. The Ca(OH)_2_ contents of samples TP0, TP20, and TP50 are 20.39%, 18.44%, and 13.66%, respectively. A total of 80% and 20% of the Ca(OH)_2_ content of sample TP0 are 16.31% and 10.20%, respectively. It is apparent that the Ca(OH)_2_ content of samples TP20 and TP50 are higher than 80% and 20% of the Ca(OH)_2_ content of sample TP0, respectively. In addition, the higher replacement ratio of ITP leads to the larger difference. The results elucidate that the addition of ITP promotes the later-age hydration of Portland cement and then generates a greater amount of Ca(OH)_2_. On the other hand, it confirms that the activity of ITP is still low at 365 days. The consumption of Ca(OH)_2_ from the pozzolanic reaction remains small. It can be seen from Figure 9a that the endothermic peak of Ca(OH)_2_ of hardened paste cured for 365 days under early high temperature curing condition also becomes weak, especially the width of the endothermic peak of sample HP50 is narrowed significantly. The Ca(OH)_2_ contents of samples HP0, HP20, and HP50 are 21.4%, 18.97%, and 12.29%, respectively. The decreasing ratios of Ca(OH)_2_ contents of cement-ITP hardened pastes cured at early high temperature are still lower than those of replacement ratios of ITP. It confirms that the reaction degree of ITP at 365 days is still low even at early high temperature curing. It is worth noting that sample HP50 shows the lower Ca(OH)_2_ content compared with sample TP50 (Figure 8b and Figure 9b). The addition of 50% ITP greatly increases the water/cement ratio. The hydration of Portland cement is dramatically promoted due to the enhanced water/binder ratio and the high temperature. The dense hydration products around the cement particle prevents the later-age hydration [33]. It also might be due to the relatively higher reaction degree of ITP at 60 °C than that at 20 °C. It also further confirms that the early high temperature curing is detrimental to the later-age hydration of composite binder containing ITP. The results are in line with the findings of non-evaporable water content (Figure 3b) and the pore structure (Figure 7).

### 3.5. BSE Images Analysis

**Figure 10:**
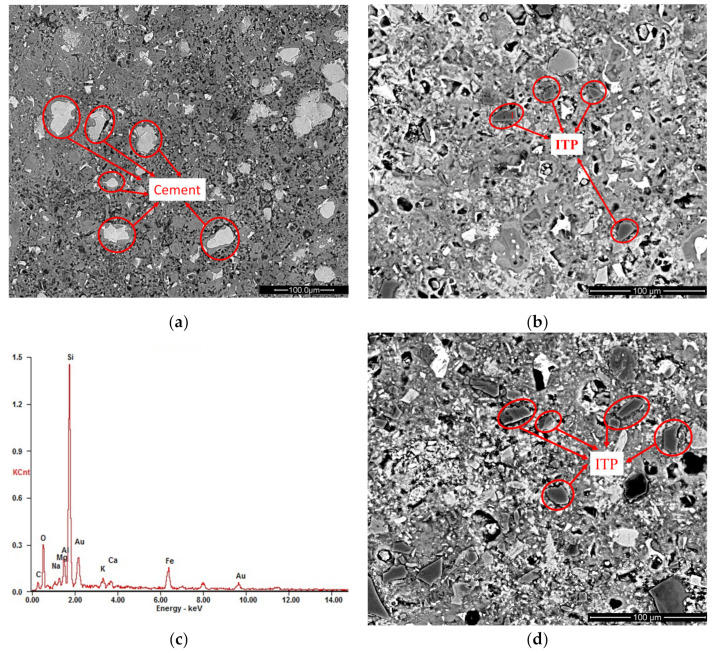
BSE images of cement-ITP hardened pastes cured for 365 days at 20 °C: (**a**) TP0; (**b**) TP20; (**c**) EDS of point “1” in Figure 11b; (**d**) TP50.

**Figure 11:**
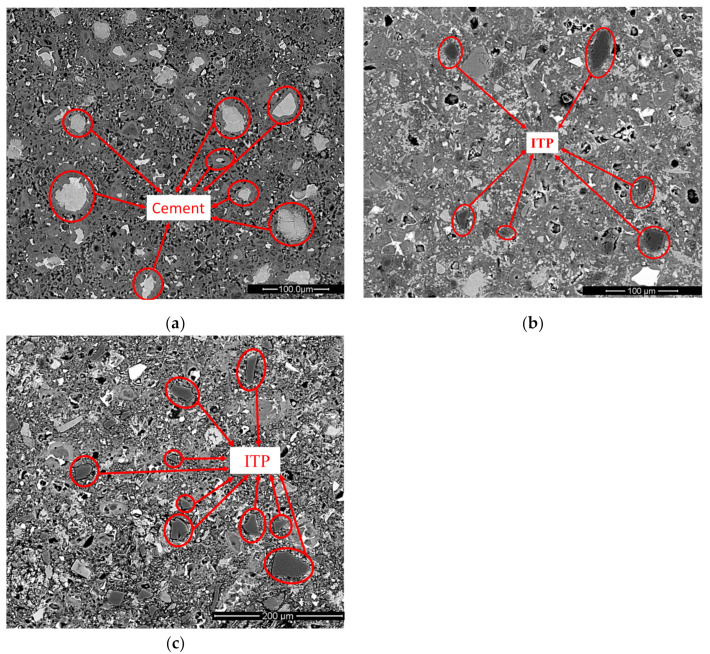
BSE images of cement-ITP hardened pastes cured for 365 days at 60 °C: (**a**) HP0; (**b**) HP20; (**c**) HP50.

Figure 10 and Figure 11 show the BSE images of cement-ITP hardened pastes cured for 365 days at 20 °C and 60 °C, respectively. Looking at Figure 10a, the small cement particles in hardened Portland cement paste have been completely hydrated. While the remaining unhydrated cement particles have larger particle sizes and are surrounded by thick layers of hydration products after curing for 365 days. It is clear that the hardened Portland cement paste cured for 365 days at 20 °C has dense structure. The quantity of unhydrated cement particles in sample TP20 is less than that in Portland cement paste (Figure 10b). The dilution effect of ITP accelerates the later-age hydration of cement. The unhydrated cement particles with large particle size can still be observed in sample TP20. The black particles in Figure 10b are ITP due to the high content of Si and Fe (Figure 10c). The large ITP particles have clear edges and angles, indicating that the large ITP particles do not react and only play a filling role in the hardened paste after 365 days. This phenomenon is more obvious in sample TP50 (Figure 10d). Many unhydrated ITP particles can be observed in sample TP50. The smaller ITP particles can be used as microaggregates, while the larger ITP particles are not closely connected with the surrounding hydration products even after hydration for 365 days, which are the weak points of the force in hardened paste. Therefore, although the addition of ITP reduces the content of large pores and the cumulative pore volume of hardened paste at 365 days (Figure 6), the interface bonding force between hardened paste and the large ITP particles is poor.

As seen in Figure 11a, the thickness of the hydration product layer around unhydrated cement particles in the Portland cement paste cured for 365 days at 60 °C is larger than that at 20 °C (Figure 10a). The distribution of hydration products is not uniform at 60 °C. The hydration products are mainly concentrated around unhydrated cement particles and the number of pores increases. Compared with the Portland cement paste, the number of unhydrated cement particles in sample HP20 is significantly reduced, and a large amount of Ca(OH)_2_ can be observed (Figure 11b). However, the edges and corners of ITP particles are still very clear, indicating that the ability of early high temperature curing to stimulate the activity of ITP is limited. The microstructure of sample HP50 cured for 365 days at 60 °C is very loose (Figure 11c). A large number of ITP particles can still be observed in sample HP50. In addition, the bond between ITP particles and the surrounding hydrates is really poor. The thermal expansion coefficient of ITP is different from that of surrounding hydration products, which leads to ITP particles peeling off from surrounding hydration products under high temperature. Then obvious gaps are observed and the coarsening pore structure is obtained (Figure 7). The weak point is formed, which is easy to cause cracks when stressed.

### 3.6. Compressive Strength

**Figure 12:**
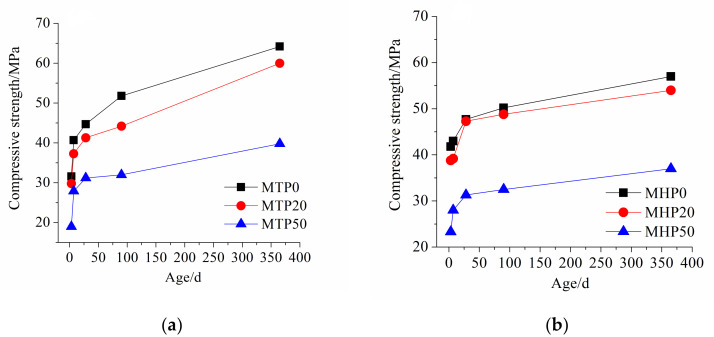
Compressive strength of cement-ITP mortar at different curing temperatures. (**a**) 20 °C and (**b**) 60 °C.

The compressive strength of cement-ITP mortar at different curing temperatures are given in Figure 12. The compressive strength of mortar decreases with increasing ITP content. As mentioned above, the activity of ITP is much lower than that of Portland cement. The early strength of mortar mainly comes from the hydration products generated by hydration of Portland cement. The addition of ITP reduces the proportion of cement in composite binder, resulting in a decrease in the amount of hydration products. Gutteridge [34,35] found that owing to the filling effect of materials, even inert materials mixed with cement would have a great influence on the hydration of cement. The physical effect of ITP (dilution and nucleation effects) can promote the hydration of cement at early age, but the physical effect of ITP cannot make up for the decrease in cement proportion. The strength difference between samples MTP20 and MTP0 is only 3 MPa at 3 days. The compressive strength is slightly decreased and the increasing rate of strength is high when 20% ITP is added. The 20% ITP addition improves the pore structure (Figure 6). However, the poor bond between ITP particles and hydration products decreases the compressive strength of mortar (Figure 10b). When 50% ITP is added, the compressive strength of mortar decreases obviously. The strength increases slowly. The low non-evaporable water content (Figure 3a) and the loose microstructure (Figure 10d) results in low compressive strength of sample MTP50.

As can be seen from Figure 12b, it is apparent that the early compressive strength increases dramatically at 60 °C. After hydration for 3 days, the strengths of MHP0, MHP20, and MHP50 cured at 60 °C are increased by 27.44%, 30.20%, and 22.63%, respectively, compared with those at 20 °C (Figure 11b). The improving effect of high temperature on early strength of the mortar containing a small amount of ITP is more significant. The increasing ratio of strength is low with increasing age. The later-age compressive strengths of all samples under early high temperature curing condition is lower than that under standard curing condition. It is indicated that the early high temperature is harmful to the later strength development of mortar containing ITP. The results are consistent with the literature [36,37,38]. The compressive strength of sample MHTP20 is very close to that of sample MHP0. However, the strength difference between samples MHP50 and MHP0 further increases at 60 °C. It is related to the lower non-evaporable water content (Figure 3b), coarse pore structure (Figure 7), and loose microstructure (Figure 11c) of sample MHP50. The early high temperature curing is detrimental to the strength development of mortar containing a large amount of ITP. Therefore, the properties of massive concrete or precast concrete containing a large amount of ITP need critical concern.

## 4. Conclusions

Increasing the amount of ITP decreases the exothermic rate and cumulative hydration heat of blended cement at 20 °C. The high temperature accelerates hydration rate and leads to the cumulative hydration heat of blended cement containing 20% ITP higher than that of Portland cement.

The non-evaporable water content decreases with increasing ITP content at both of the two temperatures. The high temperature obviously increases the early-age non-evaporable water content, but the increasing rate of non-evaporable water content is limited after 28 days.

Adding ITP coarsens the early-age pore structure, but the high temperature refines the early-age pore structure. The addition of ITP markedly improves the later-age pore structure at 20 °C. The early high temperature curing is harmful to the pore structure of hardened paste containing a large amount of ITP.

The addition of ITP decreases the Ca(OH)_2_ content. The early high temperature curing significantly reduces the Ca(OH)_2_ content of hardened paste containing a large amount of ITP at 365 days.

The reaction degree of ITP is still low at the high temperature. The large ITP particles bond poorly with surrounding hydrates. The early high temperature curing results in more loose structure of hardened paste containing a large amount of ITP.

The early high temperature significantly improves the early strength of the mortar containing a small amount of ITP, but it is detrimental to the strength development of mortar containing a large amount of ITP.

The blended cement containing ITP can be used as a binder in concrete. The properties of blended cement containing a large amount of ITP cured at high temperature require critical consideration.

## Figures and Tables

**Table 1:** Chemical compositions of raw materials (Mass/%).

Composition	SiO_2_	Al_2_O_3_	Fe_2_O_3_	CaO	MgO	SO_3_	Na_2_O_eq_	f-CaO	LOI
Cement	20.55	4.59	3.27	62.50	2.61	2.93	0.53	0.83	2.08
ITP	67.29	8.49	8.95	3.63	4.80	0.45	2.90	-	2.39

Na_2_O_eq_ = Na_2_O + 0.658 K_2_O.

**Table 2:** Mix proportions of pastes.

Sample	Water/Binder Ratio	Early Curing Temperature	Mass Fraction (%)	
			Cement	Iron Tailing Powder
TP0	0.4	20 °C	100	0
TP20			80	20
TP50			50	50
HP0	0.4	60 °C	100	0
HP20			80	20
HP50			50	50

**Table 3:** Mix proportions of mortars.

Sample	w/b Ratio	Early Curing Temperature	Mass (g)		
			Cement	ITP	ISO Standard Sand
MTP0	0.4	20 °C	450	0	1350
MTP20			360	90	
MTP50			225	225	
MHP0	0.4	60 °C	450	0	1350
MHP20			360	90	
MHP50			225	225	

## Data Availability

The data presented in this study are available on request from the corresponding author.
